# Comparing the Interobserver Reliability of 4 Methods Used to Measure Knee Laxity on Coronal Plane Stress Radiograph

**DOI:** 10.1177/23259671231215740

**Published:** 2024-01-04

**Authors:** Geoffrey W. Schemitsch, Tyler M. Hauer, Graeme Hoit, Fahad H. Al Hulaibi, Shu Yang Hu, Ali Etemad-Rezaie, Ellie B. Pinsker, Ryan M. Khan, Owen Coulter, Daniel B. Whelan

**Affiliations:** † Division of Orthopaedic Surgery, University of Toronto, Toronto, Ontario, Canada; ‡ Division of Orthopaedic Surgery, Dammam Medical Complex, Saudi Arabia; § St. Michael’s Hospital, Unity Health Toronto, Toronto, Ontario, Canada; ‖ St. Michael’s Hospital, Division of Orthopaedic Surgery, Toronto, Ontario, Canada; ¶ Kinesiology, Dalhousie University, Nova Scotia, Canada; Investigation performed at Saint Michael’s Hospital, University of Toronto, Toronto, Canada

**Keywords:** joint laxity, multiple ligament injuries, reliability, sports medicine, stress radiograph

## Abstract

**Background:**

Varus and valgus knee stress radiographs provide valuable information in the pre- and postoperative evaluation of joint laxity in patients with multiligament knee injuries (MLKIs).

**Purpose:**

To review the literature for described techniques of quantifying laxity on coronal stress radiographs of the knee and identify the most reliable method.

**Study Design:**

Cohort study (diagnosis); Level of evidence, 3.

**Methods:**

A thorough literature search using the MEDLINE and Embase databases identified 4 studies with distinct methods for objectively measuring laxity on varus and valgus stress radiographs: Heesterbeek et al (2008), Jacobsen (1976), LaPrade et al (2004), and Sawant et al (2004). To compare these methods, 200 coronal plane stress radiographs from 50 patients with MLKIs were retrospectively reviewed from an MLKI database at a single institution. The amount of varus and valgus laxity on each radiograph was measured independently by 4 reviewers using each method. Intraclass correlation coefficients (ICCs) with 95% CIs were calculated to assess the interobserver reliability of each method overall and the varus and valgus measurements individually.

**Results:**

For all 4 methods, the overall interobserver reliability was considered at least moderate. The method by Heesterbeek et al proved to have the highest interrater reliability in all domains—overall (ICC, 0.87 [95% CI, 0.85-0.90]), valgus (ICC, 0.83 [95% CI, 0.78-0.88]), and varus (ICC, 0.87 [95% CI, 0.83-0.90])—demonstrating good to excellent reliability both overall and in varus measurements and showing good reliability in valgus measurements. The method by Sawant et al demonstrated good reliability in valgus measurements. All other measures demonstrated moderate reliability.

**Conclusion:**

Available methods for measuring knee joint laxity on varus and valgus knee stress radiographs in patients with MLKIs demonstrated moderate to good interobserver reliability. The method described by Heesterbeek et al proved to have the highest reliability overall as well as in measurements on varus and valgus views individually.

Multiligament knee injuries (MLKIs) are complex orthopaedic injuries resulting from disruption to at least 2 of the major stabilizing ligamentous structures of the knee.^3,18^ These injuries are rare, representing an estimated 0.02% to 0.2% of all orthopaedic injuries.^2,3^ Clinical assessment of ligamentous laxity represents a critical step in the workup and diagnosis of MLKI, evaluation of surgical reconstruction success, and monitoring instability in conservative treatment protocols.^7,8,15^ While physical examination remains a mainstay of quantifying laxity in patients with MLKIs, it is prone to subjectivity. Stress view radiographs, however, allow visualization of laxity and the resultant joint space opening in an objective manner.

Stress view radiographs of the knee can be obtained while clinicians apply manual stress or automated force using a stress testing device in the coronal or sagittal plane.^15^ For coronal stress radiographs, the amount of medial and lateral joint space opening during valgus and varus stress testing of MLKIs, respectively, can be measured and compared with the unaffected knee. This provides clinicians with an objective and standardized evaluation of ligamentous laxity in patients with MLKIs. Any notable side-to-side differences can provide clinicians with valuable information regarding the stability of MLKIs, which can guide management and inform prognosis.^8^ Surgical indications are often based on these stress view assessments and other factors.^16^ Postoperatively, side-to-side differences can be monitored with serial stress radiographs to evaluate the integrity of operative treatment throughout the postoperative surveillance period.

There is significant heterogeneity in multiple dimensions of stress radiography—including force application, radiograph view, and joint angle.^8^ Previous authors have described varying techniques to objectively quantify joint laxity during the evaluation of valgus and varus stress radiographs.^6,7,12,17^ These techniques utilize different anatomic landmarks to measure the space or angle created between corresponding joint surfaces when valgus and varus forces are applied to the knee joint during stress radiography.^6,7,12,17^ While these methods have demonstrated strong reliability in previous studies, no investigation has compared the reliability of these measurement protocols against one another.^5,12,13^ As a result, no gold standard of varus and valgus stress radiography measurement has been identified. This has led to implementing heterogeneous methodology in clinical practice and research. Using a single measurement protocol with reliably consistent results may yield more comparable results between studies and facilitate the synthesis of the best available evidence.

The present study aimed to review the literature for described techniques of quantifying laxity on coronal stress radiographs of the knee and evaluate the accuracy and reliability of the different measurement techniques in patients with MLKIs in an effort to arrive at a recommendation for the most reliable method to use.

## Methods

### Literature Review

The study protocol received institutional review board approval. After a thorough literature search using the MEDLINE and Embase databases from inception until June 2020, we identified studies that have described 4 distinct methods for objectively measuring knee laxity on varus and valgus stress radiographs: Heesterbeek et al,^6^ Jacobsen,^7^ LaPrade et al,^13^ and Sawant et al.^17^ In the present study, these 4 studies are referred to as the Heesterbeek, Jacobsen, LaPrade, and Sawant methods. The search strategy utilized is summarized in Table 1.

**Table 1:** Search Strategy

No.	Search Terms
1	knee injuries/ or exp anterior cruciate ligament injuries/ or exp knee dislocation/
2	((multiligament* or multi-ligament*) adj1 knee adj1 (injur* or tear* or sprain* or rupture* or reconstruct* or repair* or surgery or operation*)).tw.
3	knee dislocation*.tw.
4	(multiple ligament* knee adj2 (injur* or tear* or sprain* or rupture*or reconstruct* or repair* or surgery or operation*)).tw.
5	((anterior cruciate ligament or ACL) adj2 (injur* or tear* or sprain* or rupture*or reconstruct* or repair* or surgery or operation*)).tw.
6	((posterior cruciate ligament or PCL) adj2 (injur* or tear* or sprain* or rupture*or reconstruct* or repair* or surgery or operation*)).tw.
7	((medial collateral ligament or MCL) adj2 (injur* or tear* or sprain* or rupture*or reconstruct* or repair* or surgery or operation*)).tw.
8	((lateral collateral ligament or LCL) adj2 (injur* or tear* or sprain* or rupture*or reconstruct* or repair* or surgery or operation*)).tw.
9	(posterolateral corner adj2 (injur* or tear* or sprain* or rupture*or reconstruct* or repair* or surgery or operation*)).tw.
10	(stress adj1 (radiograph* or xray* or imag* or view*)).tw.
11	((anterior or posterior or varus or valgus) adj1 stress adj1 (radiograph* or xray* or imag* or view*)).tw.
12	1 or 2 or 3 or 4 or 5 or 6 or 7 or 8 or 9
13	10 or 11
14	12 and 13

**Figure 1.**
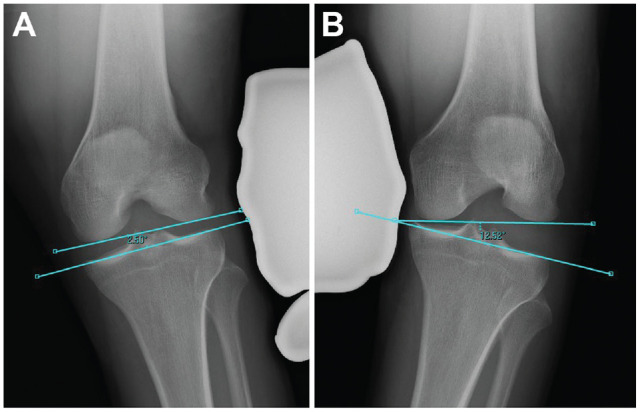
The method of Heesterbeek.^6^ The angle between a tangential line on the subchondral bone of the femoral condyle and a line through the deepest tibial plateau is measured. This value is then compared with the contralateral side. (A) Valgus measurement. (B) Varus measurement.

**Figure 2.**
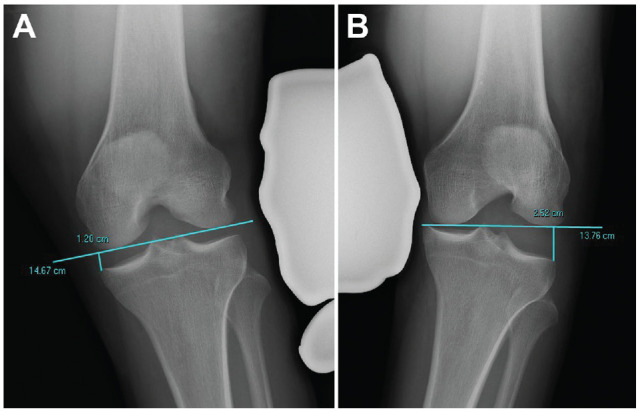
The method of Sawant.^17^ A tangential line on the subchondral bone of the femoral condyle is drawn. From this line, a perpendicular line to the most medial or lateral point of the medial or lateral tibial plateau is drawn, respectively. This value is then compared with the contralateral side. (A) Valgus measurement. (B) Varus measurement.

**Figure 3.**
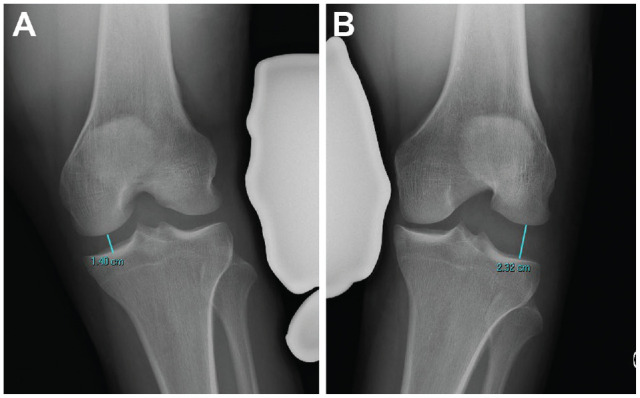
The method of LaPrade.^13^ The perpendicular distance from the subchondral bone surface of the most distal aspect of the femoral condyle and the corresponding tibial plateau is measured on both medial and lateral compartments on valgus and varus stress testing, respectively. (A) Valgus measurement. (B) Varus measurement.

**Figure 4.**
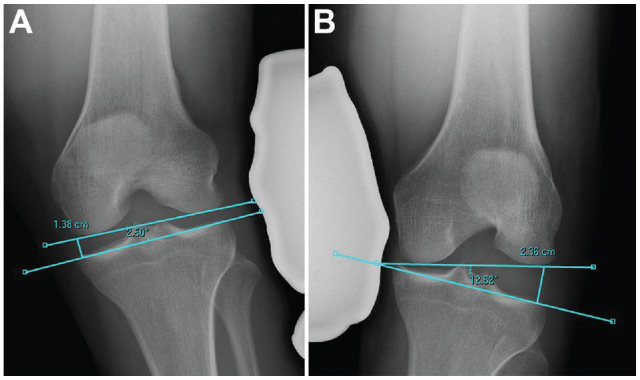
The method of Jacobsen.^7^ One line is drawn tangential to the most distal portion of the medial and lateral femoral condyle, and another line is drawn tangential to the subchondral bone of the medial and lateral tibial plateaus. Subsequently, a final line tangential to the medial and lateral border of the proximal tibial is drawn perpendicular to the tibial line measuring the distance between the 2 transverse lines for valgus and varus stress view, respectively. (A) Valgus measurement. (B) Varus measurement.

The Heesterbeek method measures the angle between a tangent line on the subchondral bone of the femoral condyles and a line between the deepest tibial joint surfaces (Figure 1). Using the Sawant method, a tangent line is drawn at the subchondral bone of the femoral condyles. From this line, the distance of a perpendicular line drawn to the most medial or lateral point of the medial or lateral tibial plateau, respectively, is recorded (Figure 2). In the LaPrade method, the distance from the most distal aspect of the subchondral bone of the medial or lateral femoral condyle to the corresponding articular surface on the tibia is recorded (Figure 3). With the Jacobson method, 1 line is drawn tangential to the most distal portion of the medial and femoral condyles, and another line tangential to the most proximal subchondral bone of the medial and lateral tibial plateaus is then drawn. One line tangential to the lateral or medial border of the proximal tibia is drawn perpendicular to the tibial line. The distance of this vertical line between the femoral and tibial horizontal lines is recorded (Figure 4).

### Radiographic Evaluation

Coronal plane stress radiographs from patients with MLKIs were reviewed from a large prospective MLKI database at a single institution. Each patient had undergone bilateral knee stress radiography with both varus and valgus stress. Measurements were completed on both injured and uninjured limbs to increase the number of radiographs reviewed and replicate the typical radiographic protocol at our institution, where the joint space opening of an injured knee is compared with the uninjured side. The maximum manual varus and valgus stress at 20° of flexion was provided by the senior author (D.B.W.) while radiographs were captured. All patients with an adequate series of bilateral postoperative coronal plane stress radiographs were considered for study inclusion. The exclusion criteria were the presence of hardware that obstructed bony landmarks and suboptimal radiograph quality. If >1 set of postoperative stress radiographs were obtained from long-term follow-ups, the most immediate postoperative radiographs were measured.

Using each method, the amount of varus and valgus laxity on each radiograph was measured once independently by 4 reviewers with different levels of experience—including a medical student (G.W.S.), a junior orthopaedic resident (T.M.H.), a senior orthopaedic resident (S.Y.H.), and a clinical fellow (F.A.H.). Each reviewer was provided with a detailed training document that defined each method. Sample radiographs labeled with measurements were provided to consolidate training. Each reviewer was given a series of radiographs to complete measurements. One reviewer was unable to complete measurements on 3 radiographs. All measurements were completed using Carestream Vue Motion Software.

### Statistical Analyses

Descriptive statistics were calculated using means and standard deviations. Intraclass correlation coefficients (ICCs) with 95% CIs were calculated based on an absolute agreement, and a 2-way random-effects model was used to assess the interobserver reliability of each method. The reliability of a single observer (ICC, 2,1) was examined. ICCs were calculated for varus and valgus measurements individually, along with a total ICC representing the pooled reliability from both valgus and varus measurements.^12^ ICCs ranged from 0 to 1, with higher ICCs indicating greater similarity between values. ICC values were interpreted according to guidelines from Koo and Li^11^: ICCs ≥0.90 indicated excellent reliability, 0.90 > ICCs > 0.75 good reliability, 0.75 > ICCs > 0.50 moderate reliability, and ICCs ≤0.50 poor reliability. All statistical analyses were conducted using SPSS Version 28.0 (IBM).

## Results

A total of 200 coronal plane stress radiographs from 50 patients with MLKIs—37 men and 13 women, with a mean (±SD) age of 44.12 ± 11.79 years—were included in the study. The mean total joint opening for all radiographs measured by the 4 observers ranged from 6.16°± 2.70° to 6.58°± 2.77° using the Heesterbeek method; 13.25 ± 3.60 mm to 16.54 ± 3.55 mm using the Jacobsen method; 12.64 ± 3.44 mm to 14.26 ± 2.66 mm using the LaPrade method; and 12.64 ± 3.44 mm to 14.26 ± 2.66 mm using the Sawant method (Table 2).

**Table 2:** Descriptive Data^*a*^

Measuring Method	Valgus (n = 97-100)	Varus (n = 97-100)	Total (n = 194-200)
Heesterbeek, deg			
Reviewer 1	5.19 ± 2.30	7.40 ± 2.56	6.29 ± 2.67
Reviewer 2	5.14 ± 2.27	7.48 ± 2.42	6.31 ± 2.61
Reviewer 3	5.08 ± 2.41	7.24 ± 2.54	6.16 ± 2.70
Reviewer 4	5.54 ± 2.56	7.63 ± 2.59	6.58 ± 2.77
Jacobsen, mm			
Reviewer 1	11.45 ± 2.70	15.04 ± 3.49	13.25 ± 3.60
Reviewer 2	14.89 ± 2.86	18.18 ± 3.43	16.54 ± 3.55
Reviewer 3	13.45 ± 2.82	16.04 ± 4.01	14.83 ± 3.55
Reviewer 4	15.04 ± 3	17.48 ± 3.59	16.26 ± 3.52
LaPrade, mm			
Reviewer 1	12.45 ± 2.06	14.90 ± 2.83	13.67 ± 2.76
Reviewer 2	13.06 ± 2.12	15.46 ± 2.62	14.26 ± 2.66
Reviewer 3	11.70 ± 2.89	13.59 ± 3.69	12.64 ± 3.44
Reviewer 4	13.14 ± 3.04	15.28 ± 2.91	14.21± 3.16
Sawant, mm			
Reviewer 1	11.75 ± 2.92	14.79 ± 3.44	13.67 ± 2.76
Reviewer 2	11.92 ± 2.76	15.41 ±3.28	14.26 ± 2.66
Reviewer 3	11.97 ± 3.07	15.22 ±3.55	12.64 ± 3.44
Reviewer 4	12.54 ± 3.36	15.39 ±3.68	14.21 ± 3.16

a Data are reported as mean ± SD.

The ICC (2,1) for each measurement method demonstrated that the Heesterbeek method showed the highest ICC (2,1) values across valgus (0.83 [95% CI, 0.78-0.88]), varus (0.87 [95% CI, 0.83-0.90]), and overall (0.87 [95% CI, 0.85-0.90]) stress test measurements (Table 3). The ICC values indicated good to excellent interobserver reliability. The Sawant method showed similar good interobserver reliability in the valgus stress test (0.82 [95% CI, 0.77-0.87]) but not in the varus (0.74 [95% CI, 0.67-0.81]) and overall stress tests (0.66 [95% CI, 0.57-0.74]). The remaining ICC values are shown in Table 3.

**Table 3:** Interrater Reliability^*a*^

Measuring Method	Valgus	Varus	Total
Heesterbeek, deg	0.83 (0.78-0.88)	0.87 (0.83-0.90)	0.87 (0.85-0.90)
Jacobsen, mm	0.60 (0.26-0.78)	0.69 (0.48-0.81)	0.72 (0.44-0.84)
LaPrade, mm	0.67 (0.55-0.76)	0.58 (0.46-0.68)	0.66 (0.57-0.74)
Sawant, mm	0.82 (0.77-0.87)	0.74 (0.67-0.81)	0.66 (0.57-0.74)

a Data are reported as ICC (2,1) (95% CI). ICC, intraclass correlation coefficients.

## Discussion

Coronal plane stress radiographs objectively measure laxity and supplement clinical examination and aid in surgical decision-making for patients with MLKIs. In this study, we evaluated 4 different measurement techniques identified in a literature review. We found that the method described by Heesterbeek et al^6^ proved to be the most reliable and accurate for quantifying medial and lateral joint laxity. However, no previous direct comparison between these methods has been conducted.

A lack of consensus in MLKI knee stress radiograph measurements has led to the implementation of heterogeneous methodology in the literature. MLKIs are rare injuries that are particularly difficult to consistently evaluate with physical examination and magnetic resonance imaging. Stress radiography provides an objective measure of joint laxity and injury stability. Previous cadaveric studies have demonstrated the potential role of stress radiography in the diagnosis of high-grade varus and valgus knee injuries.^1,13^ However, without an accepted gold standard method for measuring laxity, it is difficult to make recommendations for diagnostic values or thresholds in the workup of these patients. Identifying a single accurate measurement method would allow for threshold laxity guideline recommendations, which could facilitate decision-making in the management of these patients before surgery and during postoperative follow-ups.

Previous studies have evaluated the reliability of these methods with strong reliability. Interobserver reliability (ICCs) for the LaPrade method,^5,9^ Jacobsen method,^10,13,14^ and Sawant method^4,17^ have been reported in available studies to be 0.83 to 0.98, 0.87, and 0.95 to 0.99, respectively. The Sawant method has only been utilized to measure valgus stress radiographs in the literature. The present study demonstrated higher reliability with valgus stress using the Sawant method compared with varus stress. Furthermore, the reliability of the Heesterbeek method has not been reported in any previous research. These previously reported findings demonstrated higher reliability when compared with the present study. This may be secondary to utilizing multiple observers from multiple levels of training in the present study, which simulates the clinical environment of many academic institutions. Trainees with lower levels of orthopaedic knowledge may identify landmarks with less accuracy. In the present study, all observers underwent training before commenting on measurements to maximize their knowledge of radiographic landmarks. The Heesterbeek method demonstrated the highest reliability in all measures and may be more suitable for measuring joint laxity in future trials.

Of the 4 methods evaluated in this study, the Heesterbeek method is the only one that relies on angular measurement of medial or lateral joint space widening. In this method, tangent lines are drawn at the distal aspects of the femoral condyles and deepest portion of the tibia articular surfaces. These landmarks can be clearly identified radiographically, leading to accurate and reliable measurements of joint laxity in coronal stress radiographs. The Sawant, LaPrade, and Jacobsen methods rely on measuring vertical distance between the affected femoral condyle and the tibial plateau. This can affect the accuracy and reliability of the measurement, given the potential for variability in the form of start point, end point, and course of the vertical line measured. These techniques may be more prone to interobserver differences when there are rotational deviations in radiograph acquisition. Further research should be conducted to identify how the radiographic rotational profile affects the accuracy of these measurements. Moreover, on clinical examination, surgeons grade MKLIs according to the amount of joint space opening, similar to the Sawant, Jacobsen, and LaPrade methods, as opposed to grading injuries using angular measurements. Therefore, clinicians may gravitate toward these linear measurements, given their compatibility with physical examination findings.

### Limitations

We recognize the limitations of the present study. There was variability in devices utilized to complete measurements by the individual raters. Because of the COVID-19 pandemic, some raters completed measurements remotely, creating screen size and resolution variability. Moreover, 1 reviewer was unable to complete measurements for 3 radiographs. Nonetheless, considering the large number of radiographs measured, this would have a limited impact on the results of the present study. In addition, the Sawant method had been described from the measurement of valgus stress radiographs. Our group applied the anatomic principles of the technique to varus radiographs with comparable reliability. Including raters with varying levels of training is both a strength and a limitation of the present study. The results of the present study may be more generalizable to academic centers where medical students, surgical residents, and fellows populate the clinical environment. Last, our study did not evaluate validity. Further research is needed to correlate the degree of laxity seen with each stress technique with clinical measurements of laxity, intraoperative findings, and patient-reported outcomes to determine threshold values of clinically significant joint laxity obtained through stress radiography.

## Conclusion

Available methods for quantifying laxity on varus and valgus knee stress radiographs in patients with MLKIs demonstrated moderate to excellent interobserver reliability across the 4 methods tested. Overall, the method described by Heesterbeek et al proved the most reliable in quantifying laxity in all directions in a large prospective MLKI database. Therefore, we recommend the Heesterbeek et al method for measuring laxity on coronal plane stress radiographs. However, further research is needed to determine and compare the validity of each method.
